# Whole exome sequencing identifies genetic markers of enterovirus susceptibility in East Asians

**DOI:** 10.3389/fmicb.2024.1452595

**Published:** 2024-08-21

**Authors:** Chia-Cheng Sung, G. W. Gant Luxton, Kuo-Sheng Hung, Yung-Fu Wu, Chih-Chien Wang, Chih-Sin Hsu, Chih-Fen Hu

**Affiliations:** ^1^Department of Pediatrics, Tri-Service General Hospital, National Defense Medical Center, Taipei, Taiwan; ^2^Department of Molecular and Cellular Biology, University of California, Davis, Davis, CA, United States; ^3^Center for Precision Medicine and Genomics, Tri-Service General Hospital, National Defense Medical Center, Taipei, Taiwan; ^4^Genomics Center for Clinical and Biotechnological Applications, Cancer Progression Research Center, National Yang Ming Chiao Tung University, Taipei, Taiwan

**Keywords:** enterovirus, enterovirus infection with severe complications, whole exome sequencing, genetic signature, East Asian population

## Abstract

**Introduction:**

Following acute enterovirus (EV) infection, outcomes vary based on factors like the immune response, viral cell entry receptor expression levels, tissue tropism, and genetic factors of both the host and virus. While most individuals exhibit mild, self-limited symptoms, others may suffer severe complications or prolonged infections that can lead to autoimmune disorders.

**Methods:**

To elucidate host responses to EV infection, we performed whole exome sequencing on blood samples from both infected and uninfected individuals. Our initial focus was on genes encoding EV entry receptors—PSGL-1, SCARB2, and ANAXA2 for EV-A71, and CD155 for poliovirus—and on host genes *ACBD3* and *PI4KΒ*, crucial for EV replication.

**Results:**

Although no specific genetic variants directly associated with EV infection were identified, we discovered 118 variants across 116 genes enriched in East Asian populations through multi-layered variant filtering. These variants were further analyzed for their potential impacts on organs, biological processes, and molecular pathways. Phenome-wide association studies were conducted to refine our understanding of their contributions to EV infection susceptibility.

**Discussion:**

Our findings aim to develop a predictive panel based on these 118 variants, which could help susceptible individuals during EV outbreaks, guiding targeted clinical interventions and preventative strategies.

## 1 Introduction

Enterovirus (EV) infections are a significant global health concern, particularly affecting preschool-aged children. EVs, positive-sense single-stranded RNA viruses, are linked to diseases like epidemic pleurodynia, herpangina, and poliomyelitis (Chen et al., 2020). They spread easily via the fecal-oral route and are resistant to 75% ethanol. In Taiwan, EV outbreaks occur annually from May to September, leading to numerous illnesses and hospitalizations among young children (Wang et al., 2010). Although enterovirus infections with severe complications (EVSCs), such as acute encephalitis, are rare, they can result in significant morbidity and mortality (Lin and Huang, 2020). Between 2003 and 2018, Taiwan’s EVSC case-fatality rate ranged from 1.3 to 33.3%, with improvements due to advancements in medical care^1^.

EVs are highly cytolytic and neurotrophic, causing neuroinflammation and potential neurodegeneration. Involvement of the central nervous system can lead to neurological sequelae, delayed neurodevelopment, and impaired cognitive function (Chang et al., 2019). For example, EV-A71 can cause neurological manifestations ranging from aseptic meningitis to acute flaccid paralysis and brainstem encephalitis, which is often associated with severe pulmonary edema and shock (Solomon et al., 2010). Poliovirus, on the other hand, is a well-known cause of paralytic poliomyelitis, an acute central nervous system disease that results in flaccid paralysis (Blondel et al., 2005). Since 2013, a successful nationwide policy of non-pharmaceutical interventions in Taiwan has dramatically reduced EVSC incidence and EV-associated mortality. Despite effective vaccination against virulent EV-A71 and poliovirus, high risk serotypes like Coxsackie B, EV-D68, and echovirus still pose significant threats (Brouwer et al., 2021). In addition to the well-known neurologic sequelae caused by EV-A71 and poliovirus, Coxsackie B can lead to infections in the heart, pleura, pancreas, and liver, resulting in conditions such as pleurodynia, myocarditis, pericarditis, and hepatitis. It also causes systemic neonatal disease (Lim et al., 2013). EV-D68 has been associated with severe respiratory illness globally and has been linked to the neurological condition acute flaccid myelitis in the United States, particularly following the first outbreak in 2014 (Park et al., 2021).

Susceptibility to EV infection varies due to factors such as age, environment, season, and host genetic signatures. Some children experience recurrent infections, while others remain resistant during outbreaks. To identify genetic predispositions to EV infection in the pediatric population, we conducted whole exome sequencing (WES) of leukocytes from both infected and uninfected individuals. Initially, we examined variants in genes encoding known EV entry receptors, focusing on ANAX2, PSGL-1, and SCARB2 for EV-A71 (Yen et al., 2018), and CD155 for poliovirus (Nandi et al., 2022). However, no significant variants were identified in the exonic regions of these genes.

We then explored host genes *ACBD3* and *PI4KB*, which are critical for EV replication. EV replicates its viral genomes by remodeling the intracellular membranes of host cells and assembling viral replication organelles (ROs), which are enriched with viral replication factors and co-opted host factors. These ROs serve several important purposes during viral replication, including facilitating genome replication (Liu et al., 2023). The non-structural EV 3A protein plays a crucial role by recruiting the lipid kinase phosphatidylinositol 4-kinase IIIβ (PI4KB) to the ROs. It facilitates viral replication by binding to an essential pan-EV host factor, acyl-CoA-binding domain-containing protein 3 (ACBD3), and promoting the PI4KB-ACBD3 interaction. This interaction is important for the formation of cytoplasmic ROs, necessary for the EV lifecycle (Lyoo et al., 2019). Although discovered variants were deemed benign and clinically irrelevant, we hypothesize that additional genetic factors influence susceptibility to EV infection. We applied a meticulous, multi-layered filtering of variants and investigated the potential impacts of enriched single nucleotide variants (SNVs) on human organs, biological processes, and molecular pathways. Our goal is to evaluate these candidate SNVs for their role in EV infection susceptibility and potential contributions to disease outcomes.

## 2 Materials and methods

### 2.1 Patients and healthy controls

This study recruited 25 Taiwanese children (13 males and 12 females), aged from 1 day to 18 years, between December 2018 and February 2021 (Table 1). The participants were divided into three groups: seven children with severe infections, characterized by clinical or laboratory-confirmed EV infection involving at least one vital organ or system (e.g., central nervous system, heart, lungs, or liver); 10 children with minor infections, defined by EV infection without vital organ involvement; and eight healthy controls, all aged 7 years or older and without any history of EV infection at enrollment.

**Table 1:** General data of cases of previous enterovirus infection and healthy controls.

Subjects		Gender/Age at WES study	Age when diagnosed EV infection	Clinical presentations of EV infection	Lab data	Underlying diseases	Birth history
Cases							
Severe cases	1	M/1Y1M	1Y1M	Acute encephalitis Herpangina	Throat viral culture: EV (+), no serotype	None	Full-term
	2	F/11Y4M	11Y4M	Acute encephalitis Herpangina	Not detected by CSF/NP samples	None	Full-term
	3	M/4Y3M	8 M	Acute encephalitis Herpangina	NP EV PCR: EV (+), EV-A71	None	Full-term
	4	M/1Y6M	10 M	Acute myocarditis s/p ECMO Herpangina	NP PCR panel^1^: EV (+), no serotype; CSF PCR panel^2^: (−)	Inguinal hernia, bilateral	Full-term
	5	F/1Y	28D	Acute encephalitis HFMD	CSF EV PCR: EV (+), EV-A71	None	Full-term
	6	F/3Y	1D	Acute encephalitis/ myocarditis/hepatitis Herpangina	CSF EV PCR: EV (+), Coxsackie B1	None	Late Preterm
	7	M/1Y	1M26D	Acute encephalitis HFMD	CSF PCR panel: EV (+), Coxsackie A6	None	Full-term
Minor cases	1	F/6Y	3Y	HFMD	None	Allergic rhinitis	Full-term
	2	F/1Y	7 M	Herpangina	None	None	Full-term
	3	M/3Y	2Y	HFMD	None	Allergic rhinitis	Full-term
	4	M/3Y6M	2Y	HFMD	None	None	Full-term
	5	M/3Y	1Y6M	HFMD	None	None	Unknown
	6	F/4Y	2Y	Herpangina	None	Complex febrile seizure	Full-term
	7	M/1Y	2 M	Herpangina	NP PCR panel: EV (+), Coxsackie A10	Atopic dermatitis	Full-term
	8	M/2Y	10 M	HFMD	None	VUR, grade III	Full-term
	9	F/3Y	2Y	Herpangina	None	Constipation	Full-term
	10	F/3Y	1Y6M	Herpangina	None	None	Full-term
Healthy controls							
1		M/7Y	None	N/A	N/A	None	Full-term
2		M/18 Y	None	N/A	N/A	Frontal lobe epilepsy; ADHD	Unknown
3		F/9 Y	None	N/A	N/A	Asthma; allergic rhinitis; atopic dermatitis	Full-term
4		F/9 Y	None	N/A	N/A	Allergic rhinitis	Full-term
5		M/13Y	None	N/A	N/A	Short stature; allergic rhinitis	Full-term
6		F/15Y	None	N/A	N/A	Allergic rhinitis; bronchial asthma	Unknown
7		M/13Y	None	N/A	N/A	Migraine; allergic rhinitis;	Full-term
8		F/15Y	None	N/A	N/A	Language delay; horseshoe kidney; allergic rhinitis	Full-term

BioFire® FilmArray® Respiratory Panel. BioFire® FilmArray® Meningitis/Encephalitis (ME) Panel. Gender: M, male; F, female. Age: Y, years old; M, months old; D, days old. NP, nasopharyngeal; CSF, cerebrospinal fluid; ECMO, extracorporeal membrane oxygenation; HFMD, hand-foot-and-mouth disease; VUR, Vesicoureteral reflux; ADHD, attention deficit hyperactivity disorder; N/A, not applicable.

Since poliovirus has been eradicated in Taiwan and was certified by the World Health Organization in 2000, the government policy shifted from oral polio vaccine (OPV) to inactivated polio vaccine (IPV) in September 2011. Nowadays, all children in Taiwan are required to receive the IPV as part of the DTaP-Hib-IPV series starting at 2 months of age (DTaP-Hib-IPV at 2, 4, 6, 18 months old and booster dose of DTaP-IPV at 5 years old). Therefore, except for very young infants (under 2 months of age) such as severe cases 5, 6, and 7, all other cases received at least one does of IPV before contracting EV infection. Regarding the EV-A71 vaccines produced in Taiwan, two vaccines [MVC EV71 (Nguyen et al., 2022), EnVAX-A71 (Hung et al., 2019)] were launched in 2023, after our study and sampling period from December 2018 to February 2021. None of the subjects in our study were vaccinated with these new EV-A71 vaccines, nor did they participate in related clinical trials.

Written informed consent was obtained from all participants or their legal guardians. The study was approved by the Institutional Review Board of the Tri-Service General Hospital at the National Defense Medical Center in Taipei, Taiwan (IRB# 1–108–05-025). Clinical data were collected from corresponding clinicians and medical records.

### 2.2 Purification of genomic DNA from isolated human blood leukocytes

Genomic DNA was extracted from human leukocytes using the MagPurix® Blood DNA Extraction Kit LV and the MagPurix 24® Nucleic Acid Extraction System (Labgene Scietific®, SA, Châtel-Saint-Denis, Switzerland), following the manufacturer’s instructions.

### 2.3 WES

Human genomic DNA was sheared into approximately 150–200 base-pair fragments using a S220 Focused-Ultrasonicator (Covaris, Woburn, Massachusetts) according to the manufacturer’s instructions. Exome capture and library preparation were carried out using SureSelectXT Human All Exon V6 + UTR (Agilent Technologies, Santa Clara, CA). The prepared library was sequenced on a NovaSeq 6,000 System (Illumina, San Diego, CA) with 150 base-pair reads, producing up to 12 Gb of data per sample. Variant calling followed the Genome Analysis Toolkit (GATK) best practices for germline short variant discovery (DePristo et al., 2011). Initially, the Burrows-Wheeler Aligner (BWA) was used to align the sequenced exomes to the latest human genome reference build, hg38 (GRCh38). Duplicate reads were removed using Picard, followed by local realignment and base quality recalibration with GATK. Germline single-nucleotide polymorphisms (SNPs) and indels were identified using the GATK-HaplotypeCaller (DePristo et al., 2011).

**Figure 1:**
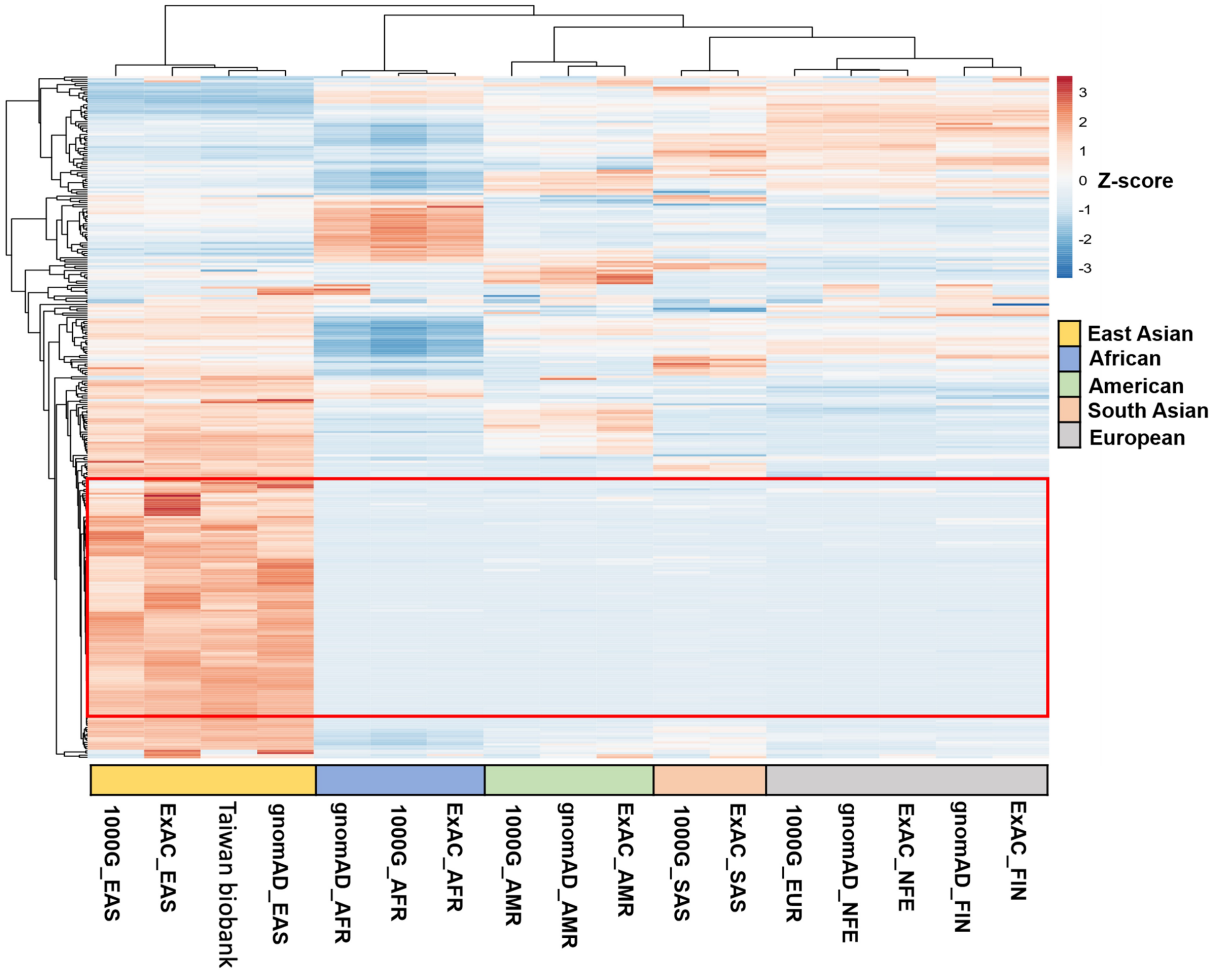
Clustered heatmaps of SNVs from hierarchical clustering analysis across different datasets. The heatmaps show hierarchical clustering of SNVs across various datasets. Of the 321 variants analyzed, 118 SNVs (highlighted in the red frame) were highly clustered in the East Asian population, in contrast to their distribution in African, American, South Asian, and European populations.

Variants were annotated using ANNOVAR (Wang et al., 2010) with databases such as refGene, clinvar_2017095, avsnp150, dbnsfp33a, and gnomAD. Annotated variants were selected based on the following criteria: located within exonic regions; non-synonymous mutations; and a read depth greater than 20 (Supplementary Figure S1). We initially examined variants in the *ACBD3, ANXA2, CD155, PI4KΒ,* and *PSGL-1* genes in both cases and controls (Supplementary Data Sheet S1). Subsequently, we filtered these variants by the following criteria: (1) an allele frequency greater than 0.01 in the East Asian population in gnomAD to exclude rare conditions; (2) potentially deleterious variants identified by SIFT = D, PolyPhen-2 = D, and CADD>20; (3) 321 variants found exclusively in EV-infected individuals (Supplementary Data Sheet S2); and (4) variants highly clustered in the East Asian population. This led to the identification of 118 (SNVs) across 116 genes (Figure 1). Variants of interest were validated through manual inspection using the Integrative Genomics Viewer.

### 2.4 Functional analysis of WES data

**Figure 2:**
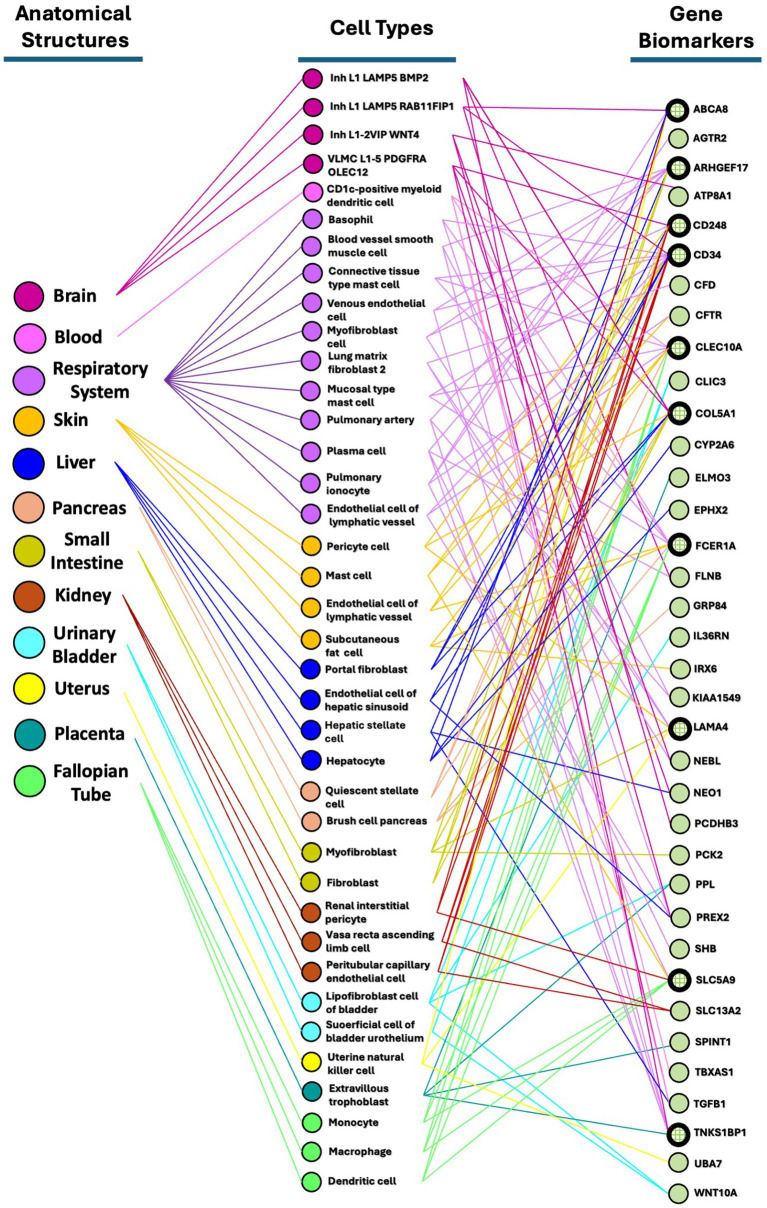
Partonomy tree showing relationships among enriched SNVs, anatomical structures, and cell types. This partonomy tree illustrates the connections between enriched SNVs or biomarkers, anatomical structures, and cell types. Among the 116 highly clustered genes in the East Asian population, 36 genes (31%) were enriched through Enrichr-KG analysis and mapped using the HuBMAP ASCTplusB Augmented 2022 dataset. This tree visualizes how anatomical structures are linked to their respective cell types and biomarkers through bimodal networks.

**Figure 3:**
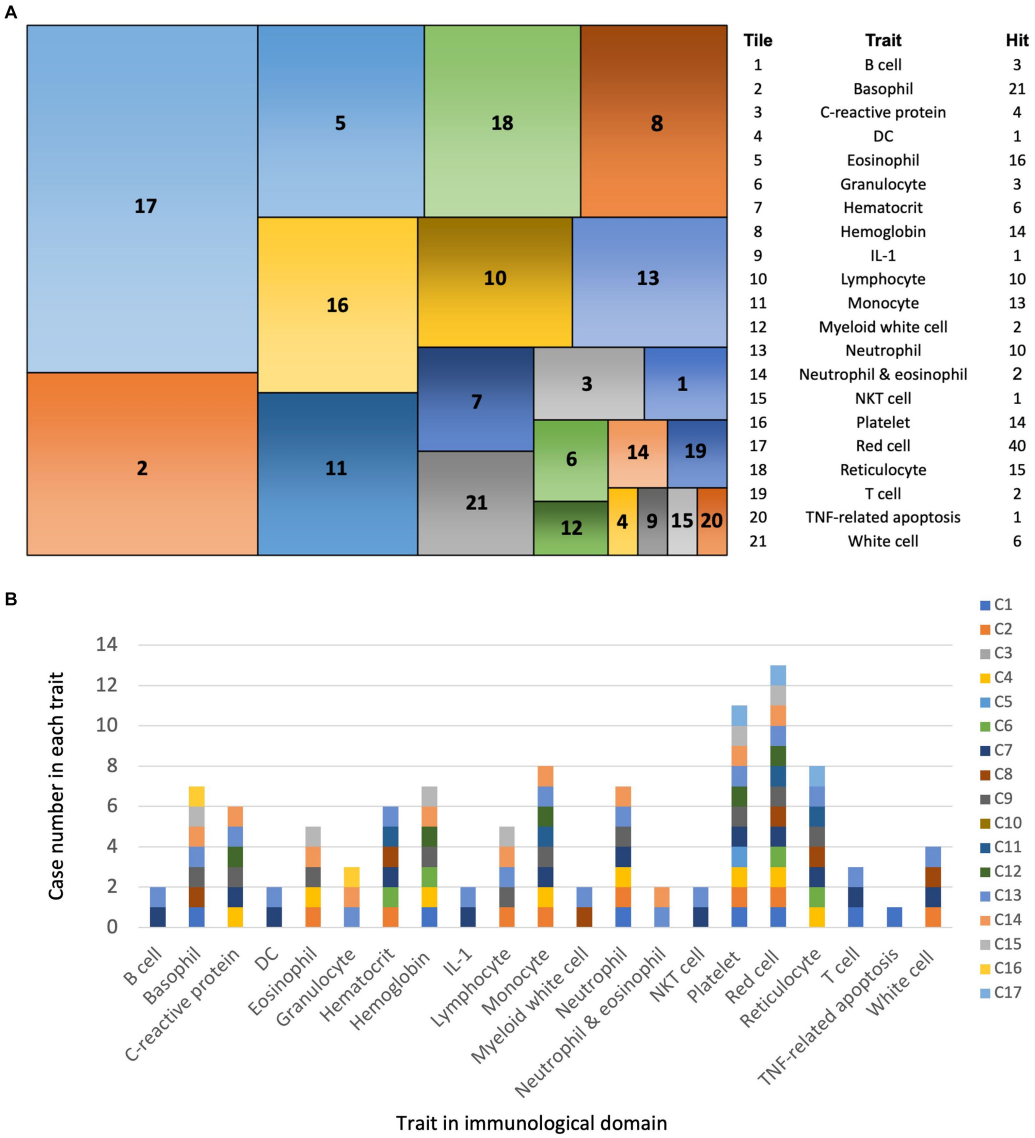
PheWAS analysis of SNVs in EV infection cases. **(A)** Tile plot: overview of phenotypes associated with SNVs from 17 EV infection cases within the immunological domain. **(B)** Bar chart: Case number of each trait among 21 immunological traits.

The final set of 118 variants across 116 genes underwent a comprehensive analysis, focusing on four main areas. Initially, tissue network analysis was performed using the Enrichr-knowledge graph (Enrichr-KG), a tool for enrichment analysis and network visualization (Evangelista et al., 2023)^2^. The enriched genes were then mapped to the 2022 augmented Human Biomolecular Atlas Program (HuBMAP) and the 2022 Anatomical Structures, Cell Types, and Biomarkers (ASCTplusB) dataset^3^ (The human body at cellular resolution, 2019). This mapping visualized the known roles of the genes in various anatomical structures, cell types, and biomarkers (Figure 2). Subsequently, gene ontology biological process analysis was conducted using Enrichr (Chen et al., 2013; Kuleshov et al., 2016; Xie et al., 2021), which mapped the genes to the GO_Biological_Process_2023 dataset^4^ (Ashburner et al., 2000; Aleksander et al., 2023) (Table 2 and Supplementary Data Sheet S3). This process provided insights into the biological processes associated with the variants. Next, molecular pathway analysis utilized Enrichr to map the genes to the Reactome_2022 pathway database^5^ (Fabregat et al., 2016), identifying relevant molecular pathways (Table 3 and Supplementary Data Sheet S4). Additionally, a phenome-wide association study (PheWAS) was performed using the genome-wide association study (GWAS) Atlas^6^ (Watanabe et al., 2019) to explore the association of these genes with various phenotypes and disease risks (Figure 3 and Supplementary Data Sheet S5).

**Table 2:** Gene Ontology (GO) biological process (BP) terms from highly clustered group in East Asian population.

Term	ID	*p*-value (<0.05)	Related genes	PubMed ID
Insulin and glucose regulation				
Positive regulation of insulin secretion involved in cellular response to glucose stimulus	GO:0035774	0.003	*CFTR; BAIAP3*	34,557,158, 37,390,839, 35,144,715
Regulation of insulin secretion involved in cellular response to glucose stimulus	GO:0061178	0.011		
Positive regulation of insulin secretion	GO:0032024	0.023		
Cellular response to glucose stimulus	GO:0071333	0.017	*NKX6-1; PCK2*	34,557,158, 37,390,839, 35,144,715
Type B pancreatic cell proliferation	GO:0044342	0.029	*NKX6-1*	34,390,364, 32,676,816
Type B pancreatic cell development	GO:0003323	0.035		
Regulation of Type B pancreatic cell development	GO:2000074	0.046		
Calcium regulation				
Regulation of release of sequestered calcium ion into cytosol by sarcoplasmic reticulum	GO:0010880	0.007	*GSTO1; DHRS7C*	35,110,649, 31,933,192
Regulation of release of sequestered calcium ion into cytosol	GO:0051279	0.036		
Positive regulation of ryanodine-sensitive calcium-release channel activity	GO:0060316	0.040	*GSTO1*	
Release of sequestered calcium ion into cytosol by endoplasmic reticulum	GO:1903514	0.040	*FASLG*	
Immune response				
Regulation of interleukin-17 production	GO:0032660	0.017	*IL23R; IL36RN*	29,549,443, 30,135,310
Positive regulation of activation of Janus kinase activity	GO:0010536	0.035	*IL23R*	34,992,585, 30,378,208 International Journal of Pediatrics. 2017 Volume 44; Issue 2, P139-141*
Regulation of activation of Janus kinase activity	GO:0010533	0.040		
Regulation of NK T cell activation	GO:0051133	0.029		
Regulation of T-helper 17 cell lineage commitment	GO:2000328	0.029		
Positive regulation of T-helper 17 cell differentiation	GO:2000321	0.035		
Positive regulation of natural killer cell proliferation	GO:0032819	0.040		
Regulation of T-helper 1 type immune response	GO:0002825	0.046		
Positive regulation of memory T cell differentiation	GO:0043382	0.046		
Positive regulation of NK T cell activation	GO:0051135	0.046		
ISG15-protein conjugation	GO:0032020	0.029	*UBA7*	35,185,830, 21,630,249
Metabolism				
Arachidonic acid metabolic process	GO:0019369	0.004	*CYP2A6; TBXAS1; CYP2F1*	19,800,403
Coumarin metabolic process	GO:0009804	0.029	*CYP2A6*	35,005,109, 32,042,967
Cyclooxygenase pathway	GO:0019371	0.046	*TBXAS1*	29,034,730, 25,890,183, 33,505,216
Cell cycle and apoptosis				
DNA damage response, signal transduction by P53 class mediator resulting in cell cycle arrest	GO:0006977	0.004	*GML; GTSE1*	35,305,353, 35,761,382, 35,510,476
Apoptotic process	GO:0006915	0.045	*CYFIP2; GML; FASLG; MCM2*	15,765,805, 28,073,399, 30,910,697, 33,481,814
Lymphocyte apoptotic process	GO:0070227	0.040	*FASLG*	16,628,611
T cell apoptotic process	GO:0070231	0.040		
Other				
Hepatocyte differentiation	GO:0070365	0.029	*PCK2*	9,128,862

* Not covered in PubMed.

**Table 3:** Reactome Pathway from highly clustered group in East Asian population.

Term	ID	P-value	Relate Genes	PubMed ID
Autophagy/Cell death signaling				
Chaperone mediated autophagy	R-HSA-9613829	0.006	*CETN1; CFTR*	31,563,390, 30,475,087, 32,943,650, 36,471,479
Selective autophagy	R-HSA-9663891	0.050		
NRAGE Signals Death Thru JNK	R-HSA-193648	0.044	*ARHGEF17; ARHGEF1*	34,502,556, 32,582,091
Death receptor signaling	R-HSA-73887	0.048	*ARHGEF17; ARHGEF1; FASLG*	3,287,048
Metabolism				
Gluconeogenesis	R-HSA-70263	0.015	*PGAM4; PCK2*	32,717,953
Vitamin C (Ascorbate) metabolism	R-HSA-196836	0.040	*GSTO1*	28,224,112
Immune				
Immune system	R-HSA-168256	0.018	CUL7; GSTO1; IL23R*; FASLG; ABL2; IFNL3	27,899,653, 27,618,897, Int. J Ped 2017 44(2):139–141*, 16,628,611, 26,729,027, 37,376,591
Other				
Regulation of gene Expression in early Pancreatic precursor cells	R-HSA-210747	0.046	*NKX6-1*	34,390,364, 32,676,816

* Not covered in PubMed.

Beyond these primary analyses, we conduced further investigations to deepen our understanding. We examined the distribution of genes across karyotypes using Ensembl (Supplementary Figure S2). To visualize the clustering of multivariate data from different geographic regions, we performed a 3D principal component analysis (PCA) using ClustVis^7^ (Supplementary Figure S3). We also used Venn diagram analysis with Interactive Venn^8^ (Heberle et al., 2015) to identify intersections of genes and SNVs between highly clustered (HC) and non-clustered (NC) groups (Supplementary Figure S4). Finally, the validation of variants of interest was carried out through manual inspection using the Integrative Genomics Viewer.

## 3 Results

### 3.1 Clinical observations

We identified 25 participants: seven individuals with severe EV infections (4 males, 3 females), 10 with minor infections (5 males, 5 females), and eight healthy controls (4 males, 4 females) (Table 1). Among the severe infection group, there were two were attributed to EV-A71, one to Coxsackie B1, one to Coxsackie A6, and two lacked further serotype verification. Acute encephalitis was the most common severe complication, occurring in six cases, followed by acute myocarditis in two cases, and acute hepatitis in one case. These critical cases exhibited varying degrees of herpangina or hand-foot-and-mouth disease, in addition to symptoms and signs involving vital organs. Severe infections were confined to children under 1 year of age (newborn and infant stages), while mild infections occurred in children aged 2 months to 3 years. Cases not classified as severe or mild were complicated by significant prenatal, birth histories, or underlying diseases. There were no fatalities in the severe infection group, and these individuals displayed acceptable milestone development at the time of blood sampling for this WES study. However, two cases experienced long-term central hypoventilation that needed tracheostomy and intermittent assisted ventilation (severe case no. 2 and 3), and one exhibited unilateral limb weakness as sequelae (severe case no. 2).

### 3.2 Assessment of genetic variants in EV-A71 and poliovirus receptor-encoding genes and their impact on host susceptibility to EV infection

We initially examined genetic variants in known EV-A71 entry-related receptors—ANXA2, PSGL-1, and SCARB2 (Yen et al., 2018)—among our cases and healthy controls. Additionally, we analyzed the poliovirus receptor CD155 (Nandi et al., 2022), which facilitates poliovirus binding, entry into susceptible cells, and regulation of the cell-mediated immune system. No variants were found in the exonic regions of the genes encoding these receptors in either group.

Next, we investigated the genes encoding ACBD3 and PI4KΒ, as mutations in these genes could potentially disrupt their interaction and consequently affect EV replication. For ACBD3, we identified two SNVs: rs145831627 in severe case no. 1 and rs2306120 in severe case no. 7. For PI4KB, we found one SNV, rs28372941, in a healthy control no. 3 (Supplementary Data Sheet S1). These variants are predicted to be benign and tolerable, with no clinical relevance. This suggests that additional genetic variants may influence host susceptibility to EV infection and disease progression.

### 3.3 Genetic comparison of combined severe and minor EV infection cohorts to healthy controls

Our search for genetic signatures that differentiate the severe infection group from the minor infection group did not reveal any definitive candidate variants. Consequently, we combined the severe and minor infection groups into a single cohort of 17 cases and compared this to a group of 8 heathy controls.

#### 3.3.1 Distinct genetic patterns in East Asians revealed by clustering analysis of EV infection-associated

Following our WES analysis workflow (Supplementary Figure S1), 321 variants were found exclusively in EV-infected individuals (Supplementary Data Sheet S2). We then utilized clustered heatmaps of SNVs from hierarchical clustering analysis across different datasets in different geographic regions. Of the 321 variants analyzed, 118 SNVs were highly clustered in the East Asian population, in contrast to their distribution in African, American, South Asian, and European populations. This led to the identification of 118 SNVs across 116 genes (Figure 1) as our candidate list. Initially, we assessed the distribution of these SNVs across the 23 pairs of human chromosomes and mitochondrial DNA. The SNVs were evenly distributed from chromosomes 1 to X, with no variants found on chromosome Y or in mitochondrial DNA (Supplementary Figure S2).

To visualize the clustering of multivariate data from different geographic regions, we employed 3D PCA. This method enabled us to assess the clustering of datasets from East Asia, Africa, America, South Asia, and Europe, using genomic databases such as gnomAD, Taiwan Biobank, ExAC, and the 1,000 Genomes project. The datasets include East Asian (gnomAD_EAS, Taiwan Biobank, ExAC_EAS, 1000G_EAS), African (gnomAD_AFR, ExAC_AFR, 1000G_AFR), American (gnomAD_AMR, ExAC_AMR, 1000G_AMR), South Asian (ExAC_SAS, 1000G_SAS), and European populations (gnomAD_FIN, ExAC_FIN, gnomAD_NFE, ExAC_NFE, 1000G_EUR). Firstly, we applied 3D plot that combines principal components PC1, PC2, and PC3 to provide a comprehensive view of the data distribution. Next, we applied 2D plots with pairwise comparisons of the principal components—PC1 vs. PC2, PC2 vs. PC3, and PC1 vs. PC3, respectively in a plan view. The 3D scatter plot revealed distinct clusters for populations from the same geographic region and clear separation between different regions (Supplementary Figure S3), indicating unique genetic signatures in East Asian populations. Consequently, we analyzed our WES data using the gnomAD_EAS dataset, as it provides a representative reference for the East Asian population.

We then examined the overlap of genes and SNVs between the HC group in the East Asian population (116 genes and 118 SNVs) and the NC group (184 genes and 203 SNVs). No overlapping genes or SNVs were found between the HC and NC groups (Supplementary Figure S4), demonstrating that the HC group’s genetic variants are distinct from those in the NC group.

In summary, the 118 SNVs enriched in the HC group are unique to the East Asian region and clearly distinguishable from other geographic regions due to the lack of overlap with the NC group. Our findings were categorized into four main areas: tissue network analysis (Figure 2), gene ontology biological process analysis (Table 2 and Supplementary Data Sheet S3), molecular pathway analysis (Table 3 and Supplementary Data Sheet S4), and GWAS analysis in PheWAS (Figure 3 and Supplementary Data Sheet S5).

#### 3.3.2 Mapping genetic variants associated with EV infection to tissue networks and cell types in East Asians using HuBMAP

We used Enrichr-KG to perform enrichment analysis with various gene set libraries and mapped the results to the augmented HuBMAP ASCTplusB 2022 dataset (The human body at cellular resolution, 2019). This dataset enables the creation of tissue maps and an atlas of cell functions and relationships within the human body. Our goal was to capture the hierarchical structure of human anatomical parts, cell types, and biomarkers, including genes, proteins, lipids, or metabolic markers. We generated bimodal tissue networks to connect anatomical structures to cell types and cell types to biomarkers among these highly clustered genes.

From the 116 highly clustered genes in the East Asian population, 36 (31%) were successfully mapped in the HuBMAP ASCTpluB dataset. This mapping identified 12 anatomical structures associated with 38 cell types. Notably, the respiratory system was linked to 11 cell types associated with EV infection: basophils, blood vessel smooth muscle cells, connective tissue type mast cells, venous endothelial cells, myofibroblasts, lung matrix fibroblasts type 2, mucosal mast cells, pulmonary artery cells, plasma cells, pulmonary ionocytes, and lymphatic vessel endothelial cells. The brain was connected to four cell types (Inh L1 LAMP5 BMP2, Inh L1 LAMP5 RAB11FIP1, Inh L1-2 VIP WNT4, VLMC L1-5 PDGFRA OLEC12), and the blood system to one (CD1c-positive myeloid dendritic cells). The skin had four cell types (pericytes, mast cells, lymphatic vessel endothelial cells, subcutaneous fat cells), the liver had four (portal fibroblasts, hepatic sinusoid endothelial cells, hepatic stellate cells, hepatocytes), and the pancreas had two (quiescent stellate cells, pancreatic brush cells). The small intestine had two cell types (myofibroblasts, fibroblasts), the kidney had three (renal interstitial pericytes, vasa recta ascending limb cells, peritubular capillary endothelial cells), and the urinary bladder had two (bladder lipofibroblasts, superficial cell of bladder urothelium). The uterus was linked to one cell type (uterine natural killer cells), the placenta to one (extravillous trophoblasts), and the fallopian tubes to three (monocytes, macrophages, dendritic cells).

Among the 36 identified gene biomarkers, the top 10 most frequently associated with anatomical structures and cell types were: *CD34* (14 associations), *ARHGEF17* (10), *CLEC10A* (10), *COL5A1* (10), *FCER1A* (10), *CD248* (9), *LAMA4* (6), *SLC5A9* (6), *TNKS1BP1* (6), and *ABCA8* (5) (Figure 2). *CD34*, hematopoietic progenitor cell antigen, may play a role in the attachment of stem cells to the bone marrow extracellular matrix or to stromal cells (Radu et al., 2023) and is involved in pathways such as the innate immune system as well as class I MHC mediated antigen processing and presentation. *CLEC10A* (Jondle et al., 2016) and *FCER1A* (Tang et al., 2023) are involved in immune responses, while *ARHGEF17*, *COL5A1*, and *CD248* have no apparent roles in the immune system.

#### 3.3.3 Gene ontology analysis reveals biological processes linked to increased EV infection susceptibility in East Asia

We used Enrichr to map our results onto the GO_Biological_Process_2023 dataset (Ashburner et al., 2000; Aleksander et al., 2023) to identify the biological processes, cellular components, and molecular functions involved in gene ontology analysis. This analysis focused on genes differentially expressed between cases and controls. In gene ontology terms, a biological process involves all the steps necessary to achieve a specific biological objective, executed through a series of regulated molecular functions.

Our analysis revealed that 59 of the 116 genes (51%) were associated with 87 biological processes (*p* < 0.05). Among these, 18 genes (16%) were linked to 30 biological processes specifically referenced in EV infection. Notably, these included processes related to the immune response, which genes such as *IL23R*, *IL36RN*, and *UBA7*; insulin and glucose regulation, involving genes like *CFTR*, *BAIAP3*, *NKX6-1*, and *PCK2*; and calcium regulation, with genes *GSTO1*, *DHRS7C*, and *FASLG*. Additionally, processes associated with the cell cycle and apoptosis were highlighted, involving genes *CYFIP2*, *GML*, *FASLG*, *MCM2*, and *GTSE1.* Metabolic processes were linked to genes *CYP2A6*, *TBXAS1*, and *CYP2F1*, while a single process was categorized as “other,” involving gene *PCK2* (Table 2 and Supplementary Data Sheet S3).

The findings underscore the significant role of immune response processes during EV infection, suggesting that regulation of insulin and glucose might be related to diabetes mellitus (DM), specifically type 1 DM (Morse et al., 2023). Furthermore, the involvement in calcium regulation, cell cycle, and apoptosis indicates that EVs might exploit these cellular mechanisms to aid in their viral entry, replication, maturation, and release.

#### 3.3.4 Reactome analysis reveals pathways linked to increased susceptibility to EV infection in East Asia

We then used Enrichr to map our results to the Reactome_2022 dataset (Fabregat et al., 2016). In Reactome, the fundamental unit of data is the reaction, which includes various entities such as nucleic acids, proteins, complexes, vaccines, anti-cancer therapeutics, and small molecules. These entities participate in a network of biological interactions grouped into pathways. This framework aids in interpreting high-throughput experimental results and developing algorithms for extracting insights from genomic data.

Our mapping indicated that 39 of the 116 genes (34%) were involved in 31 pathways (*p* < 0.05). Among these, 13 genes (11%) were linked to 8 pathways specifically referenced in EV infection. Notably, four pathways were related to autophagy and cell death signaling, involving genes (*CETN1*, *CFTR*, *ARHGEF17*, *ARHGEF1*, and *FASLG*). Two pathways were associated with metabolism, involving genes *PGAM4*, *PCK2*, and *GSTO1.* One pathway was linked to the immune response, involving genes *CUL7*, *GSTO1*, *IL23R*, *FASLG*, *ABL2*, and *IFNL3*, while one pathway was categorized as “other,” involving the gene *NKX6-1* (Table 3 and Supplementary Data Sheet S4).

In addition to the pathways directly related to EV infection, the dysregulation of autophagy and cell death signaling may play a significant role in the pathogenesis following EV entry. EVs exploit autophagy to create membrane-bound ROs necessary for viral RNA replication and virion maturation. They may also arrange autophagic vesicles for viral particle assembly or release and use these processes to evade the host immune response (Zhang et al., 2020).

#### 3.3.5 PheWAS analysis reveals genetic associations with immunological traits and EV infection susceptibility in East Asians

PheWAS reverse the approach of GWAS by starting with specific SNVs or genes and exploring their associations across a broad spectrum of human phenotypes, collectively known as the “phenome” (Bastarache et al., 2022). PheWAS uses genetic variants or genes as a starting point and scans a curated set of phenotypes from large patient cohorts to identify associations. Developed with data from electronic health records linked to DNA databases, PheWAS offers insights into how genetic variations correlate with diverse phenotypic outcomes across human populations.

Using the GWAS Atlas-PheWAS tool (Watanabe et al., 2019) (see text footnote 6), we identified 39 genes associated with 21 traits within the immunological domain. The most frequently associated traits were red blood cell (RBC, 40 associations), basophils (21 associations), and eosinophils (16 associations) (Figure 3A). If we sum up all the hit number from each case, there were between 1 and 55 hit number among the 21 traits (Figure 3B). Among the EV infection cases, there were between 1 and 42 associations in the PheWAS, with the exception of cases 3 and 10, which showed no associations (Supplementary Data Sheet S5).

Significantly, RBCs, the most commonly associated trait, may act as immune sensors. When bound to numerous inflammation-causing nucleic acids, RBCs can lose their normal structure, leading to their removal from circulation through macrophage erythrophagocytosis. This process can trigger immune responses in otherwise unaffected organs, resulting in inflammation (Lam et al., 2021).

## 4 Discussion

Most EV infections are mild or asymptomatic, particularly in adults (Chen et al., 2020). However, certain high-risk EV serotypes, such as EV-A71, EV-D68, Coxsackie B, and echovirus, can be virulent and aggressive in some individuals (Brouwer et al., 2021). EV-A71, first isolated in California in 1969, has been linked to outbreaks of encephalitis and aseptic meningitis (Schmidt et al., 1974). Since then, several EV-A71 epidemics with high mortality rates have occurred in the U.S., Europe, and especially in East Asia (Lee and Chang, 2010; Bubba et al., 2020). Following a severe EV outbreak with high mortality in Taiwan in 1998, the Taiwanese government established robust diseases surveillance systems for ongoing monitoring of EV epidemiology. Although vaccines for EV-A71 are now available (Hung et al., 2019; Li et al., 2021; Nguyen et al., 2022), challenges remain from other high-risk serotypes. The extensive availability of human genetic and genomic information enables the investigation of host susceptibility to EV infections, allowing for the identification of vulnerable individuals and effective prevention of EV infections and severe complications. This proactive approach aims to prevent long-term sequelae, such as type 1 DM, an autoimmune disease that destroys insulin-producing pancreatic beta cells (Morse et al., 2023).

In our workflow (Supplementary Figure S1), we identified 118 SNVs across 116 genes specific to EV cases in East Asia. Using Enricher for gene set enrichment analysis, we mapped these genes to the HuBMAP ASCTpluB dataset to determine which organs, systems, and cell types are involved during EV infection. The respiratory system emerged as the most affected, followed by the digestive system, including the liver, pancreas, and small intestine. The most frequently identified cell types were immune-related hematopoietic cells, such as CD1c-positive myeloid dendritic cells, and endothelial cells in various organs such as venous endothelial cells. The most recurrent gene biomarker was *CD34,* typically representing hematopoietic stem/progenitor cells (Radu et al., 2023). Gene ontology analysis also showed that genes such as *IL23R, IL36RN,* and *UBA7* participate in 11 immune response-related processes. Similarly, the Reactome pathway analysis highlighted roles for six genes—*CUL7*, *GSTO1*, *IL23R*, *FASLG*, *ABL2*, and *IFNL3*—in the immune system. Take *GSTO1* and *IFNL3* for examples, Liu et al. (2016) studied the immunogenicity of VP1 subunit from EV-A71 in murine macrophage RAW264.7 cells, noting a decrease in the expression of GSTO1, an immune system processing related protein. Wells et al. (2022) established an *in vivo* mouse model of echovirus infection via the enteral route and defined the roles of the type III interferons, IFNL2 and IFNL3, in controlling viral replication in the intestine using murine-derived primary enteroids.

Acute EV infection is influenced by the host immune system, the expression of viral entry receptors, tissue tropism, and the genetics of both the host and the virus. EV infection induces an innate immune response followed by an adaptive immune response, crucial for viral clearance (Bopegamage, 2016). There is growing evidence that EV can trigger autoimmune responses (Morse et al., 2023). Among the genes mapped to gene ontology biological processes and Reactome pathways, *IL23R* is linked to axial spondylarthritis, inflammatory bowel disease, and psoriasis (Brown et al., 2019), while *IL36RN* is associated with psoriasis (Liu et al., 2020). Research by Ruohtula et al. (2020) suggests that EV infection is associated with down-regulation of *FOXP3* expression in regulatory T cells (Tregs), essential for maintaining immune homeostasis and preventing autoimmune diseases (Ruohtula et al., 2020). This down-regulation leads to increased Th1 immunity, promoting proinflammatory reactions and potentially perpetuating autoimmune responses (Ruohtula et al., 2020). Consequently, lower *FOXP3* expression in Tregs after EV infection may impair the suppression of antiviral responses and tissue inflammation, contributing to autoimmunity.

Our analysis using the human GWAS Atlas for phenotype-associated SNVs in the immunological domain identified the top three disease traits as RBCs (40 associations), basophils (21 associations), and eosinophils (16 associations). Combining RBC-related SNVs with those associated with reticulocytes (15 associations), hematocrit (6 associations), and hemoglobin (14 associations), we found that 75 out of 185 associations (40.5%) were related to RBCs. These cells are critical mediators of the innate immune system, capable of binding inflammatory molecules such as chemokines, nucleic acids, and pathogens (Anderson et al., 2018). Despite lacking a nucleus and the ability to perform transcription or translation, RBCs can serve as chemokine reservoirs, releasing or removing them in response to inflammation. Hemoglobin and heme also trigger the production of reactive oxygen species to eliminate pathogens, promoting inflammation and autoimmune responses (Anderson et al., 2018). RBCs can bind bacterial DNA, malaria parasite-derived mitochondrial DNA, and synthetic CpG DNA. During sepsis, plasma levels of CpG DNA rise significantly. RBCs bind excess CpG DNA via toll-like receptor 9 (TLR9), leading to morphological changes and erythrophagocytosis by macrophages, causing anemia and systemic inflammation (Minton, 2021). Reports have documented hemolytic anemia following EV infection, manifesting as autoimmune hemolytic anemia (Pattanakitsakul et al., 2022) or atypical hemolytic uremic syndrome (Lee et al., 2013) in children. In such cases, immunosuppressants are recommended.

Immune-related hematopoietic cells and endothelial cells in different tissues play crucial roles in the real-time detection and prevention of EV infection. If the host fails to eradicate EV from organs or systems, the virus may cause chronic or latent infections, accelerating the onset of autoimmune diseases, especially type 1 DM (Richardson and Morgan, 2018). In humans, several epidemiological, clinical studies, and experimental data, strongly support the involvement of enteroviruses, and in particular Coxsackie B, with the appearance of islet autoantibodies and an increased risk of T1DM (Nekoua et al., 2022).

Beyond immune and autoimmune responses, enriched analysis of gene ontology biological processes identified several other biological processes linked to EV infection susceptibility. The second most enriched process involved insulin and glucose regulation, with genes *CFTR*, *BAIAP3*, *NKX6-1*, and *PCK2* correlating with an increased risk of type 1 DM following EV infection. Other significant processes included calcium regulation, involving genes *GSTO1*, *DHRS7C*, and *FASLG*, and processes related to cell cycle and apoptosis, involving genes *CYFIP2*, *GML*, *FASLG*, *MCM2*, and *GTSE1*.

Calcium ions (Ca^2+^) are essential for viral entry, gene replication, maturation, and release (Chen et al., 2019). During the replication cycle, viruses disrupt host Ca^2+^ channels or pumps, affecting cellular Ca^2+^ homeostasis. Evidence suggests that the EV 2B protein reduces Ca^2+^ content in the endoplasmic reticulum and Golgi apparatus, inhibiting protein trafficking and perturbing Ca^2+^ signaling (van Kuppeveld et al., 2005) To prevent early termination of the viral cycle, EVs have developed anti-apoptotic mechanisms. EVs may inhibit apoptosis by modulating the PI3K/Akt signaling pathway and/or the autophagy pathway. In the late stages of infection, EVs regulate apoptosis and host translation, aiding virus release and spread (Lai et al., 2020).

Finally, our analysis identified five genes involved in autophagy and cell death signaling: *CETN1*; *CFTR*, *ARHGEF17*, *ARHGEF1*, and *FASLG*. EVs exploit autophagic machinery to counter host antiviral defenses, facilitating viral replication, maturation, and assembly. This process ensures that only RNA-loaded virions are packaged in phagophores, segregating them from other autophagic cargo (Huang and Yue, 2020). EVs may spread via autophagosome-mediated exit without cell lysis (Huang and Yue, 2020). Each EV family member uses distinct strategies to subvert autophagy for viral benefit (Lai et al., 2016; Huang and Yue, 2020).

In summary, EVs utilize calcium regulation, apoptosis, and autophagy to enhance their replication and spread. These processes could serve as potential therapeutic targets for treating and preventing EV infections.

## 5 Conclusion

EV infection in children remains a significant global concern, particularly in Asia (Chen et al., 2020). While vaccines for poliovirus and EV-A71 are available, strategies are still needed to address threats from other high-risk EV serotypes. To advance precision medicine and improve patient care, we propose using the gene and SNV lists enriched from the East Asian dataset to differentiate between EV-susceptible and -resistant individuals. Our ultimate goal is to develop a predictive panel of genetic variants tailored for preventive medicine in EV infection. By identifying genetic markers associated with EV susceptibility, we aim to enable targeted interventions and proactive measures to reduce the impact of the disease. This approach holds promise for personalized healthcare strategies, improving treatment outcomes and public health efforts.

## Data availability statement

The data presented in the study are deposited in the NCBI BioProject repository, accession number PRJNA1145565.

## Ethics statement

The studies involving humans were approved by the Institutional Review Board of the Tri-Service General Hospital at the National Defense Medical Center in Taipei, Taiwan (IRB# 1-108-05-025). The studies were conducted in accordance with the local legislation and institutional requirements. Written informed consent for participation in this study was provided by the participants’ legal guardians.

## Author contributions

C-CS: Conceptualization, Data curation, Formal analysis, Investigation, Methodology, Writing – original draft. GL: Supervision, Validation, Visualization, Writing – review & editing. K-SH: Data curation, Formal analysis, Investigation, Methodology, Software, Validation, Writing – original draft. Y-FW: Data curation, Formal analysis, Investigation, Methodology, Writing – original draft. C-CW: Funding acquisition, Project administration, Resources, Supervision, Writing – review & editing. C-SH: Conceptualization, Data curation, Visualization, Writing – original draft. C-FH: Conceptualization, Funding acquisition, Investigation, Methodology, Project administration, Resources, Supervision, Validation, Writing – review & editing.

## Footnotes

^1^ https://www.cdc.gov.tw

^2^ https://maayanlab.cloud/Enrichr

^3^ https://hubmapconsortium.github.io/ccf-asct-reporter/

^4^ https://geneontology.org

^5^ https://reactome.org

^6^ https://atlas.ctglab.nl/

^7^ https://biit.cs.ut.ee/clustvis/

^8^ http://www.interactivenn.net
