# Supplementary figures and images for: Whole exome sequencing identifies genetic markers of enterovirus susceptibility in East Asians
Source: Front Microbiol. 2024 Aug 21;15:1452595. doi: 10.3389/fmicb.2024.1452595 (PMC11372244; doi:10.3389/fmicb.2024.1452595)

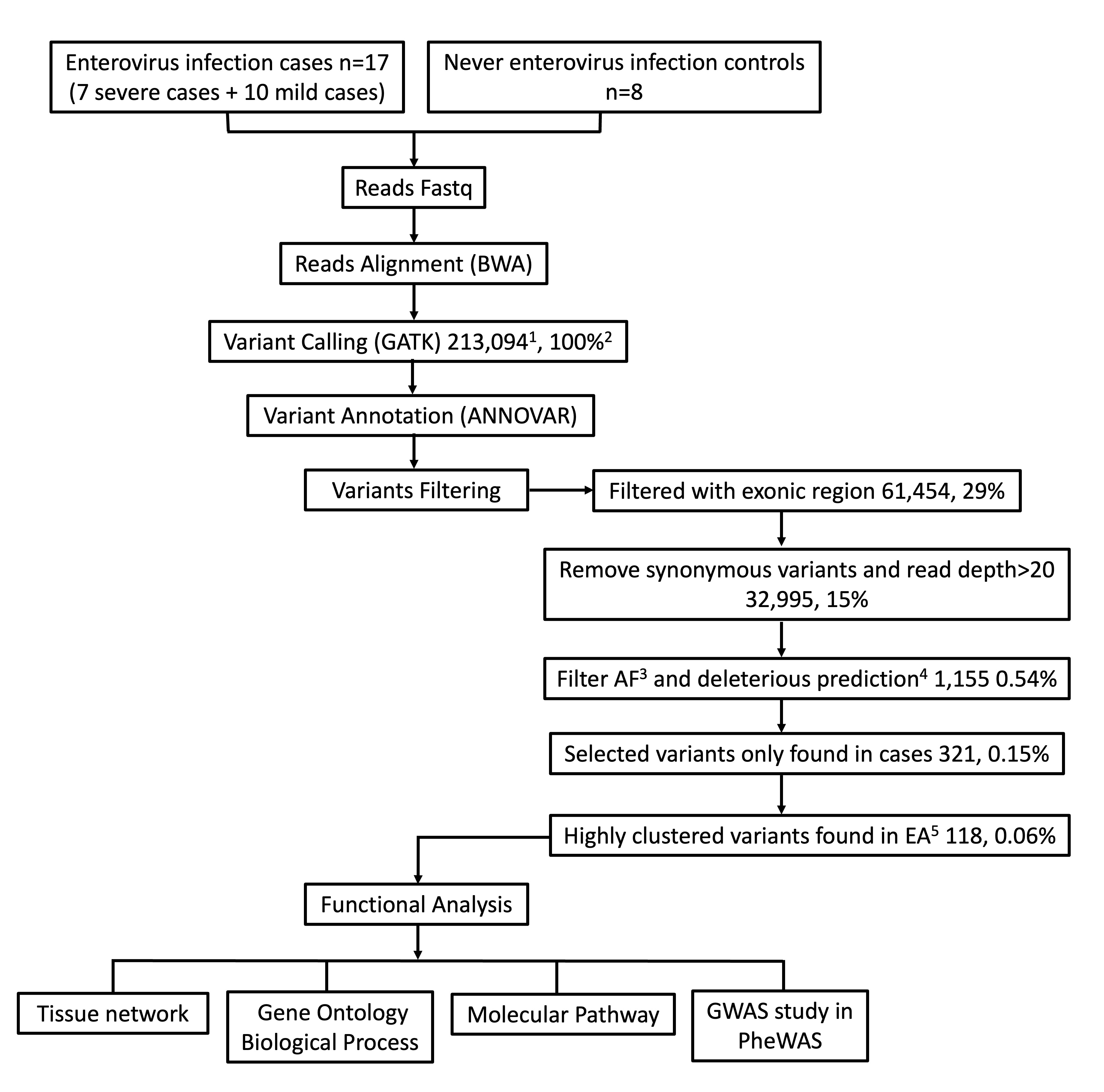

Supplement: Supplementary file 6 [file Image_1.JPEG]

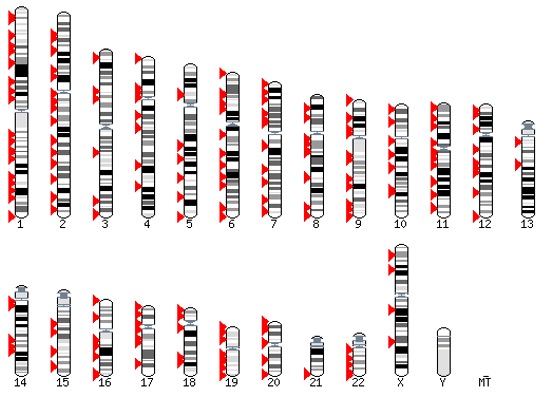

Supplement: Supplementary file 7 [file Image_2.JPEG]

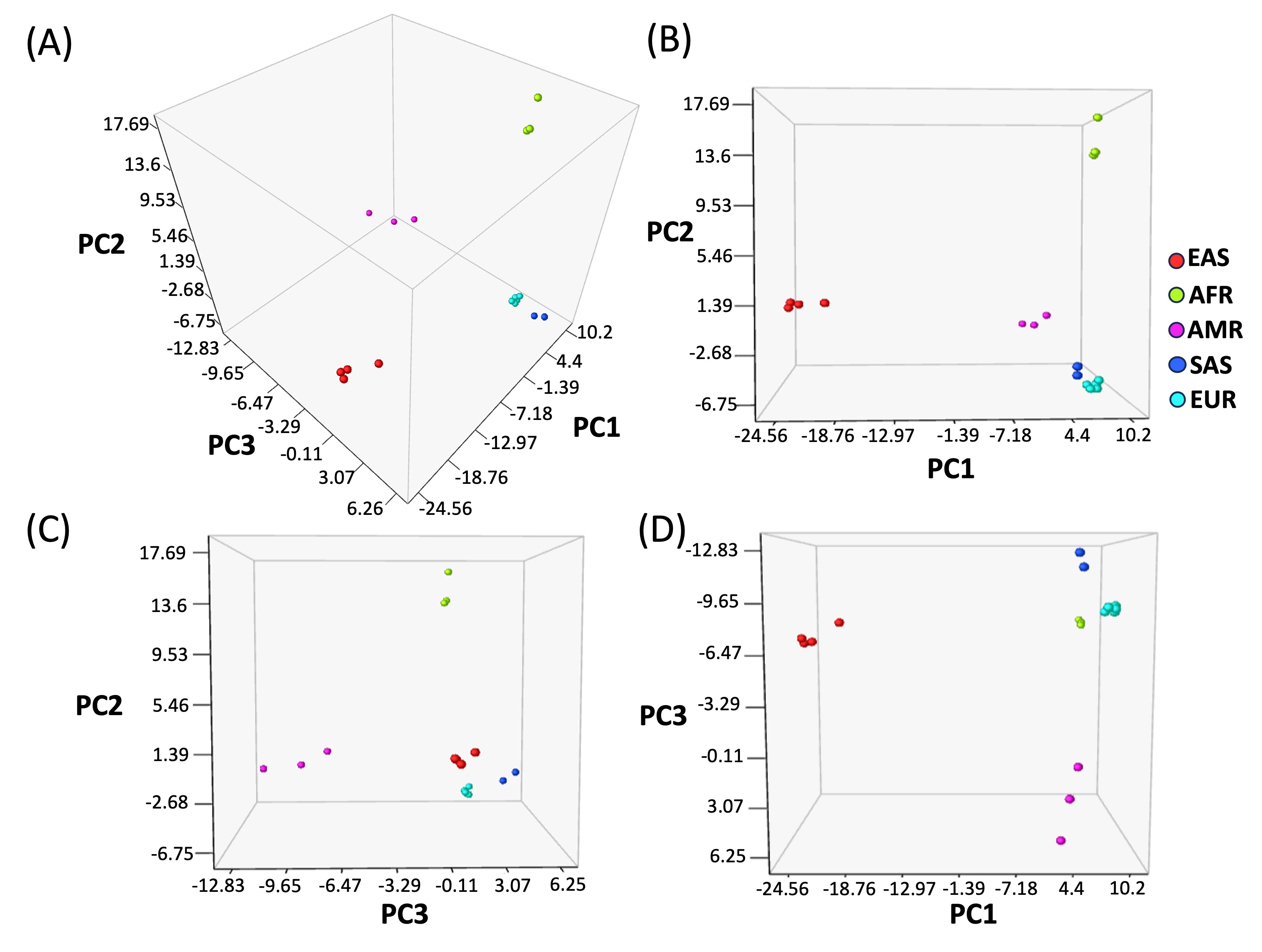

Supplement: Supplementary file 8 [file Image_3.JPEG]

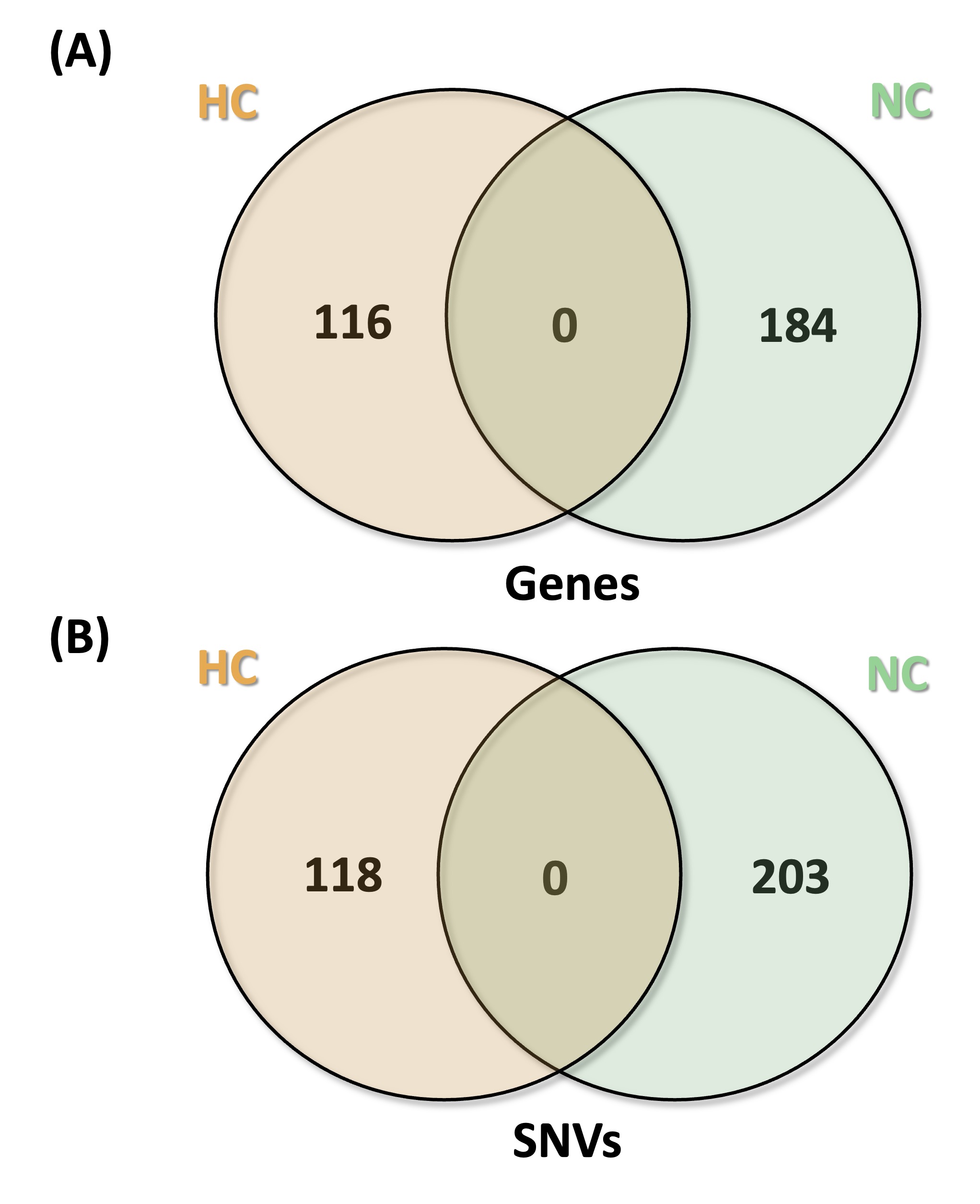

Supplement: Supplementary file 9 [file Image_4.JPEG]
